# Spatio-temporal dynamics and laterality effects of face inversion, feature presence and configuration, and face outline

**DOI:** 10.3389/fnhum.2014.00868

**Published:** 2014-11-10

**Authors:** Ksenija Marinkovic, Maureen G. Courtney, Thomas Witzel, Anders M. Dale, Eric Halgren

**Affiliations:** ^1^Department of Radiology, University of California San DiegoLa Jolla, CA, USA; ^2^Department of Psychology, San Diego State UniversitySan Diego, CA, USA; ^3^Cognitive Neuroimaging Laboratory, Center for Memory and Brain, Boston UniversityBoston, MA, USA; ^4^Athinoula A. Martinos Center for Biomedical Imaging, Massachusetts General Hospital, Radiology Department at Harvard Medical SchoolBoston, MA, USA; ^5^Department of Neurosciences, University of California San DiegoLa Jolla, CA, USA

**Keywords:** magnetoencephalography, faces, fusiform gyrus, temporal cortex, laterality, dual route model, face inversion

## Abstract

Although a crucial role of the fusiform gyrus (FG) in face processing has been demonstrated with a variety of methods, converging evidence suggests that face processing involves an interactive and overlapping processing cascade in distributed brain areas. Here we examine the spatio-temporal stages and their functional tuning to face inversion, presence and configuration of inner features, and face contour in healthy subjects during passive viewing. Anatomically-constrained magnetoencephalography (aMEG) combines high-density whole-head MEG recordings and distributed source modeling with high-resolution structural MRI. Each person's reconstructed cortical surface served to constrain noise-normalized minimum norm inverse source estimates. The earliest activity was estimated to the occipital cortex at ~100 ms after stimulus onset and was sensitive to an initial coarse level visual analysis. Activity in the right-lateralized ventral temporal area (inclusive of the FG) peaked at ~160 ms and was largest to inverted faces. Images containing facial features in the veridical and rearranged configuration irrespective of the facial outline elicited intermediate level activity. The M160 stage may provide structural representations necessary for downstream distributed areas to process identity and emotional expression. However, inverted faces additionally engaged the left ventral temporal area at ~180 ms and were uniquely subserved by bilateral processing. This observation is consistent with the dual route model and spared processing of inverted faces in prosopagnosia. The subsequent deflection, peaking at ~240 ms in the anterior temporal areas bilaterally, was largest to normal, upright faces. It may reflect initial engagement of the distributed network subserving individuation and familiarity. These results support dynamic models suggesting that processing of unfamiliar faces in the absence of a cognitive task is subserved by a distributed and interactive neural circuit.

## Introduction

Faces have captured a great deal of attention in the neuroimaging field, resulting in important insights into the brain networks that underlie material-specific processing. Based on neuroimaging evidence of right-dominant activity in the fusiform cortex that is greater to faces than other meaningful visual stimuli, this area has been termed the “fusiform face area” (Kanwisher et al., 1997; Kanwisher and Yovel, 2006), although the nature of its “face-specificity” has been debated (Gauthier et al., 1999; Halgren et al., 2000; Haxby et al., 2001; Haxby, 2006; Cowell and Cottrell, 2013).

Studies using temporally precise methodology such as ERPs (Event-Related Potentials) and MEG (Magnetoencephalography) reveal a face-sensitive deflection peaking at around 170 ms (N170 and its magnetic counterpart M170) estimated to that region (Lu et al., 1991; Halgren et al., 2000; Liu et al., 2000; Watanabe et al., 2003; Schweinberger et al., 2007; Eimer, 2011; Miki et al., 2011; Rossion and Jacques, 2011; Taylor et al., 2011). Intracranial studies confirm both the timing and the location of the primary generator of these potentials in the inferotemporal region (Allison et al., 1994; Halgren et al., 1994a; McCarthy et al., 1997; Puce et al., 1997; Barbeau et al., 2008) but also indicate that the face processing is subserved by a distributed network additionally comprising anterior temporal and prefrontal regions (Halgren et al., 1994b; Klopp et al., 1999; Marinkovic et al., 2000; Barbeau et al., 2008). Generators of face-induced N170 are highly consistent with the fMRI activity in the right fusiform gyrus (FG) (Puce et al., 1997) although fMRI studies also confirm engagement of distributed occipital, temporal, and frontal areas (Ishai et al., 2004; Chan and Downing, 2011).

Converging evidence suggests that faces are processed in a series of successive, but overlapping and mutually interactive processing stages engaging multiple brain areas. Following encoding in the posterior visual areas (at ~100 ms), activation peaks in the FG at about 170 ms after stimulus onset. At this time it is briefly phase locked with the activity in distributed association cortices primarily in ventral temporal and prefrontal regions (Klopp et al., 2000), suggesting that the face processing is mediated by a network of simultaneously active sources during the N170 stage. The N170 is followed by a deflection at ~240 ms (Barbeau et al., 2008) and subsequent activity that mediates integration with mnemonic, emotional, and other contributions in distributed areas, resulting in face recognition (Halgren et al., 1994a,b; Puce et al., 1999). This broad outline of the spatio-temporal activity pattern is consistent with the original model proposed by Bruce and Young (1986) which, in turn, serves as the foundation of the currently prevalent accounts (Halgren et al., 1994a; Haxby et al., 2002; Ishai, 2008; Behrmann and Plaut, 2013). Even though these models conceptualize face processing as being mostly sequential in nature, it is clear that this is an interactive process with overlapping, rather than discrete and temporally circumscribed stages (Halgren et al., 1994a,b; Barbeau et al., 2008; Behrmann and Plaut, 2013). They flexibly mediate structural encoding, familiarity, and retrieval of semantic information resulting in recognition, with an increasing degree of reliance on distributed and interactive circuits.

The goal of this study was to examine the spatio-temporal stages and the functional tuning of the areas engaged during face processing with an anatomically-constrained MEG method. This multimodal methodology combines whole-head high-density MEG and a distributed source modeling approach with high-resolution structural MRI and cortical reconstruction to estimate the anatomical distribution of the underlying neural networks in a time-sensitive manner (Dale and Sereno, 1993; Hämäläinen and Ilmoniemi, 1994; Dale et al., 1999, 2000; Fischl et al., 1999a). Our analysis focused on both the relative amplitudes and latencies of the deflections evoked by faces and other conditions, as well as the spatial pattern of estimated activation. In particular, we wished to examine the sensitivity of the M170 to presence and configuration of inner features, face inversion, and face outline. Some of these variables have been manipulated in other studies (Bentin et al., 1996; Eimer, 2000b; Tong et al., 2000; Macchi Cassia et al., 2006; Zion-Golumbic and Bentin, 2007; Harris and Nakayama, 2008; Rossion and Jacques, 2008; Liu et al., 2010; Nichols et al., 2010; Gao et al., 2013) but we aimed to explore these effects in a more comprehensive manner. We used grayscale photographs of unfamiliar faces and manipulated face orientation (upright vs. inverted), internal features and external outline (present or absent) and the relative feature configuration (canonical vs. rearranged) resulting in the following conditions: “normal—N,” “inverted—I,” correct facial features presented in an oval without the hairline (“oval—O”), unnaturally rearranged facial features within the natural face outline (“rearranged—R”), blank faces with natural outlines but with no features (“blank—B”). Visual control (C) stimuli were obtained by randomizing grayscale patches of the face images so that they no longer looked like faces while preserving the spatial frequency, luminance, and overall shape. We were especially interested in investigating the functional profile of the M170 and its sensitivity to the presence and absence of features and their arrangement. For instance, if it indeed reflects a face-encoding stage, then it will be responsive to the presence of facial features irrespective of the facial outline (Bentin et al., 1996; Eimer, 2000b; Tong et al., 2000; Zion-Golumbic and Bentin, 2007). Furthermore, by using methodology that provides reasonable spatial source estimates, we wished to examine the spatial characteristics of the M170. For instance, even though the right hemisphere (RH) dominance of the M170 has been established (Halgren et al., 2000; Rossion et al., 2003a; Kloth et al., 2006), contributions of the left hemisphere (LH) at this latency in the context of these manipulations are not clear.

A special case is presented by inverting face stimuli and we included this condition in our study. Impaired recognition of faces that are presented upside-down, relative to other objects (Valentine, 1988) has been termed the “face inversion effect” and is associated with larger amplitude and longer latency of the N170 (Rossion et al., 1999; Eimer, 2000a; Itier and Taylor, 2004a). fMRI studies, however, show that the inverted faces evoke either a smaller or equivalent activity in the FG than the upright faces (Kanwisher et al., 1998; Gauthier et al., 1999; Haxby et al., 1999). Moreover, some fMRI evidence suggests that inverted faces also recruit non-face (“object”) areas, evoking stronger responses more medially (Aguirre et al., 1999; Haxby et al., 1999). The dual route model suggests that inverted faces are additionally processed by the LH in a feature-based manner (Moscovitch et al., 1997; De Gelder and Rouw, 2001). This model was examined by comparing the M170 activity to inverted faces in the left and right fusiform cortices and in other engaged areas. The M170 is commonly followed by activity peaking at ~240 ms which is the earliest deflection that is reliably sensitive to face repetition and may reflect emergence of familiarity through learning (Tanaka et al., 2006; Schweinberger et al., 2007; Zimmermann and Eimer, 2013). We examined spatio-temporal characteristics of the M240 and its activity profile as a function of face orientation, features, and outline. Given that our primary focus of interest was the M170 and the relatively early processing stages that are relevant to the stimulus manipulations, we wished to minimize the semantic aspects of the processing. To that end, we used faces that were unfamiliar to our participants and employed a task of passive viewing with short presentation intervals.

## Methods

### Subjects

MEG recordings and structural MRI scans were obtained from 14 healthy right-handed male subjects between 22 and 29 years of age (mean = 24.21 ± 1.85). The subjects had no neurological impairments and no structural brain abnormalities were seen on their MRI scans. All subjects signed statements of consent that were approved by the relevant review board and were monetarily reimbursed for their participation.

### Material

**Figure 1:**
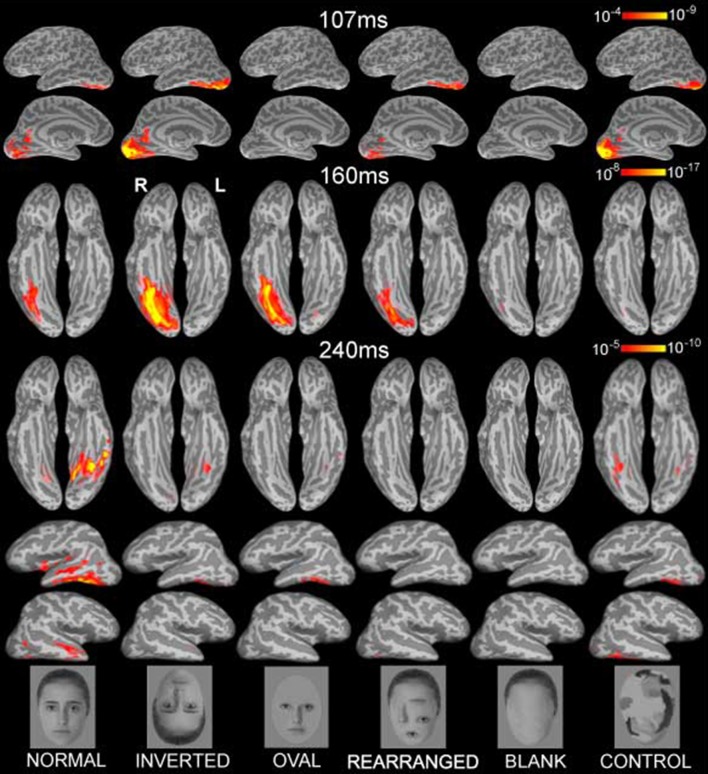
**Group-based average dynamic statistical parametric maps of estimated activity to all six conditions on ventral surfaces at ~107 ms, ~160 ms, and at ~240 ms, showing estimates in ventral and lateral views**. Early visual activity (at ~107 ms) is stronger to inverted faces and control stimuli. Inverted faces evoked the strongest M160 activity estimated to the fusiform gyrus, followed by oval, normal and rearranged images. Blank faces and randomized control faces evoked the weakest activity. The subsequent deflection, peaking at ~240ms was largest to normal faces in the ventral and anterolateral temporal areas bilaterally. Examples of the images are shown below. The individual in the photos consented to the publication of these images.

Participants viewed six different types of grayscale photos (examples are shown in Figure 1) including: normal upright faces (N), inverted faces (I), normal face features presented in an oval without hairline (O), faces with features that were rearranged into unnatural positions (R), blank faces without features but with normal hairline (B), and randomized visual control stimuli (C). The control stimuli consisted of random grayscale patches that no longer looked like faces but that preserved the spatial frequency, luminance, and overall shape. In an effort to ascertain that image manipulations did not cause potentially confounding changes in visual properties, a 2D spatial FFT was calculated across images. The control stimuli did not differ from normal faces in the mean power at low, middle or high spatial frequency bands (<5, 5–15, or 15–40 cycles per degree of visual angle, respectively). The stimulus set was comprised of the photos of six different Caucasian individuals that were not familiar to any of our subjects. All faces had neutral expression and were selected from a larger set used in prior studies (Marinkovic and Halgren, 1998). The six photographs were manipulated to obtain images across all six conditions.

### Task

During the MEG recording session the subjects were instructed to passively observe images that were presented in a randomized order on a computer-driven back-projection screen in front of the subject. Each image was presented for 225 ms at 1 s intervals on a gray background within a visual angle subtending 4° horizontal × 6° vertical. Each stimulus was repeated 16 times, yielding a total of 96 stimuli per condition.

### Data acquisition and analysis

MEG signals were recorded from 204 channels (102 pairs of planar gradiometers) with a whole-head Neuromag Vectorview instrument (Elekta Neuromag) in a magnetically and electrically shielded room. The signals were recorded continuously with 601 Hz sampling rate and minimal filtering (0.1–200 Hz). Averages for each stimulus type were constructed from trials free of eyeblinks or other occasional artifacts. On average, 8.5 ± 4.4% trials were discarded. The position of magnetic coils attached to the skull, the main fiduciary points such as the nose, nasion and preauricular points, as well as a large array of random points spread across the scalp were digitized with 3Space Isotrak II system for subsequent precise co-registration with structural MRI images.

Each person's cortical surface was reconstructed from high-resolution T1-weighted MRI structural images (1.5T Picker Eclipse, Marconi Medical, Cleveland OH) and was subsampled to ~2500 dipole locations per hemisphere (Dale et al., 1999; Fischl et al., 1999a). This cortical surface served as the solution space to constrain a noise-normalized minimum norm inverse solution, here termed anatomically-constrained MEG or aMEG. The forward solution was calculated using a boundary element model (Oostendorp and Van Oosterom, 1991). Using a linear estimation minimum norm approach with no constraints on dipole orientation (Dale and Sereno, 1993; Hämäläinen and Ilmoniemi, 1994), dipole strength power was estimated at each cortical location every 5 ms. The estimates were normalized by noise obtained from the average pre-stimulus baseline which reduced the point-spread function variability (Liu et al., 2002a), and resulted in a series of frames of dynamic statistical parametric maps (dSPMs) of estimated cortical activity (Dale et al., 2000). These noise-normalized estimates of the current dipole power for each location fit the F distribution and can be viewed as “brain movies” as they unfold in time. Group averages for each condition were obtained by aligning cortical folding patterns across all individuals and averaging their inverse estimates (Fischl et al., 1999b; Dale et al., 2000). Figure 1 presents the group average dSPMs of the overall activity patterns evoked by each stimulus condition at 107, 160, and 240 ms after stimulus onset. Estimated cortical activity is displayed on inflated views of an averaged cortical surface.

Whereas the movie snapshots represent estimated activity for the whole cortical surface at each time point, an alternative way of examining the data is to look at the timecourses (estimated noise-normalized dipole strength across time) for the selected regions of interest (ROIs). These waveforms represent estimated dipole strength moments in the cortical *source* space and are suitable for assessing the effects of stimulus conditions on both amplitude and latency (Marinkovic et al., 2003). In order to further explore activity timecourses and to ascertain statistical significance of the particular comparisons, ROIs were chosen for the relevant areas on the cortical surface based on the overall group average estimated activity. They included the posterior occipital cortex (Occ), the lateral FG, and ventrolateral anterior temporal cortex (aTL). The same group-based ROIs were used for all subjects in a manner blind to their individual activations by means of an automatic spherical morphing procedure (Fischl et al., 1999b). The ROIs contained 4.8 ± 2.3 vertices on average, corresponding to ~2.7 cm^2^ of the cortical surface. The noise-normalized dipole strength estimates were averaged across all cortical points contained in each ROI at each time point. These values obtained for each subject and task condition were used for the statistical analysis. Within-subject ANOVAs were employed to examine differences in activity among conditions at different latencies. In most cases it was possible to determine singular amplitude peaks within the three latency windows of interest. For the occipital activity peaking at ~107 ms (M107), peak amplitudes were detected within a 90–125 ms time window for each subject and task condition with an automatic algorithm. This made it possible to also examine task condition effects on peak latencies. Similarly, peak amplitude of the M160 in the right FG was identified within 130–190 ms time window for each subject and task condition. Activity in the left hemisphere at this latency was weaker and less consistent across subjects, making it difficult to detect amplitude peaks. Instead, average amplitudes were used to examine task condition effects on the activity within the 120–150 and 170–190 ms latency windows in the left FG. Within-subject ANOVA (Woodward et al., 1990) was used to statistically compare differences across conditions for each ROI and each of the three deflections. The Bonferroni method (Woodward et al., 1990) was used as a conservative protection against inflated *p*-values due to multiple comparisons and the adjusted *p*-values are reported unless specified differently.

## Results

**Figure 2:**
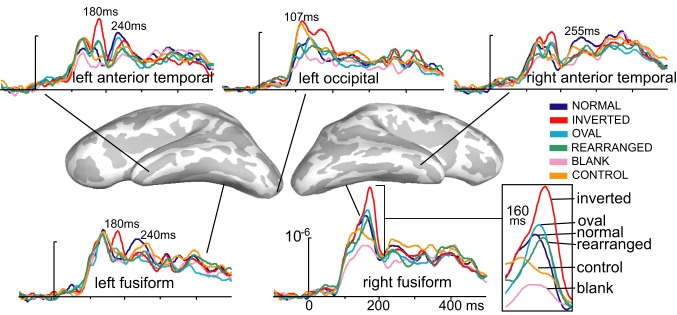
**Group-based average time courses of the estimated noise-normalized dipole strengths to all six conditions in selected cortical locations**. The earliest activity was estimated to the occipital region at ~107 ms after stimulus onset and was strongest to inverted and control images. At ~160 ms, inverted faces elicited the strongest activity in the right-lateralized ventral temporal area, centered on the fusiform gyrus. Canonically oriented stimuli with inner features irrespective of their arrangement elicited identical activity at ~160 ms on the right. Inverted faces additionally elicited the immediately subsequent deflection at ~180 ms on the left. The M240 was largest to normal, upright faces in the anterior temporal areas bilaterally, possibly reflecting the initial engagement of the network subserving individuation, acquired familiarity, and recognition.

**Figure 3:**
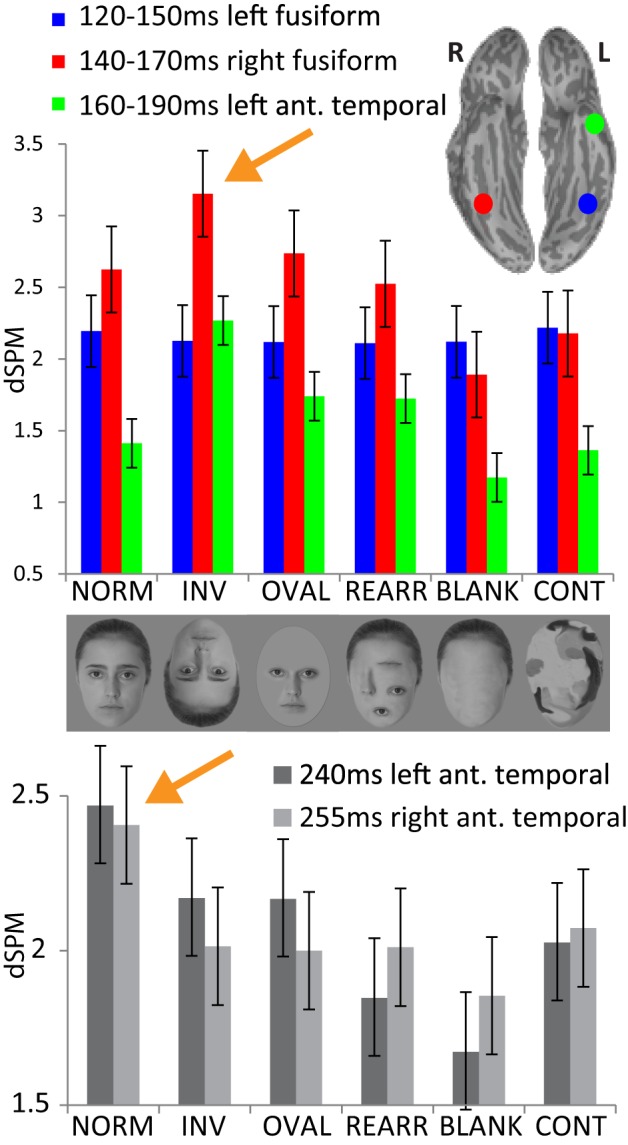
**Upper panel: group average noise-normalized dipole strengths expressed as dSPM F-values for the ROIs in the fusiform cortex bilaterally and in the left anterior temporal area representing the successive processing stages: no activity difference in the left fusiform 120–150 ms across conditions; strongest activity to inverted faces in the right fusiform (140–170 ms) and in the left anterior temporal area (160–190 ms)**. Lower panel: noise-normalized peak amplitude dipole strength estimates in the anterior temporal areas bilaterally within 220–270 ms. Normal, upright faces elicit the strongest activity in both hemispheres, possibly reflecting acquired familiarity processing.

Inspection of the overall activity indicates that the earliest activity is estimated to the occipital region at ~107 ms (M107) after stimulus onset. It propagates anteriorly via the ventral visual stream to the predominantly right ventral temporal areas peaking at ~160 ms (M160), and further on to the anterior ventrolateral temporal and prefrontal regions at ~240 ms (M240). Group-average dSPM estimates are shown in Figure 1 for the activity at 107, 160, and 240 ms. Timecourses derived from the relevant ROIs are shown in Figure 2, and graphs of mean estimated activity across all conditions in Figure 3. Table 1 summarizes main results across the ROIs and peak latencies.

**Table 1:** **ANOVA results for the main effects and condition contrasts carried out for M107, M160, and M240 response amplitudes and peak latencies**.

**ROI**	**Hemi**.	**Avg. lat**.	**Measure**	**Lat. range**	**m.e. *F*_(5, 65)_**	***p*-value**	**Contrast**	***F*_(1, 13)_**	**Bonf. *p***
**M107**									
Occ	Both	107	Peak amp.	90–125	8.0	0.0001	I > all	14.9	0.01
							C > all	7.8	0.075
		107	Peak lat.	90–125	3.4	0.009	I > all	11.1	0.05
**M160**									
FG	RH	160	Peak amp.	130–190	8.4	0.0001	I > all	14.8	0.01
							B < all	21.5	0.01
FG	LH	140	Avg. amp.	120–150	0.1	0.1			
		180	Avg. amp.	170–190	2.5	0.05			
aTL	LH	180	Avg. amp.	170–190	9.6	0.001	I > all	22.5	0.005
**M240**									
FG	LH	240	Peak amp.	210–250	2.5	0.05	N > all	8.4	0.061
aTL	LH	240	Peak amp.	210–250	3.2	0.01	N > all	11.4	0.05
aTL	LH	240	Peak lat.	210–250	2.6	0.05			
aTL	RH	255	Peak amp.	220–270	1.9	0.1	N > all	9.8	0.05

The p-values for condition contrasts are reported with Bonferroni adjustment.

The early occipital response peaks at 107 ms with a very similar amplitude and profile in both hemispheres. This observation was confirmed with an ANOVA of the peak amplitude (within 90–125 ms timewindow) with the factors of hemisphere and condition type. There was no main effect of laterality [*F*_(1, 13)_ = 0.29, *p* > 0.5] and no laterality x condition interaction [*F*_(5, 65)_ = 0.99, *p* > 0.45], so the results were pooled across both hemispheres. The main effect of Condition [*F*_(5, 65)_ = 8.0, *p* < 0.0001] results from a greater peak amplitude to inverted faces and control stimuli [*F*_(1, 13)_ = 12.9, *p* < 0.05] as compared to all other stimuli. The peak latency (107 ms) does not differ between the two hemispheres, *F*_(1, 13)_ = 0.61 *p* > 0.45), but the peak latency to inverted faces (111 ms) is longer than the latency to all other stimuli (106 ms), *F*_(1, 13)_ = 11.1, *p* < 0.05.

The subsequent deflection (M160) is right-dominant and is estimated to the fusiform cortex (Figure 1). ANOVA of the peak amplitude (within 130–190 ms time window) indicates that the right M160 is uniquely sensitive to condition differences as shown by the significant main effect, *F*_(5, 65)_ = 8.4, *p* < 0.0001. Inverted faces evoke the greatest activity amplitude than all other stimuli, *F*_(1, 13)_ = 14.8, *p* < 0.01, followed by other stimuli that include facial features such as the oval, normal, and rearranged faces (Figures 1–3). Activity to normal, upright faces does not differ from the activity to faces with rearranged features, or to normal features presented in an oval. That is, the canonically oriented stimuli containing inner features regardless of their arrangement elicit activity that appears to be very similar at ~160 ms latency. Blank facial outlines with no features elicit the weakest activity, *F*_(1, 13)_ = 21.5, *p* < 0.01.

At around this latency, activity estimated to the left FG is much weaker overall (Figures 1, 2). Since the peak patterns at this latency in the left hemisphere are not consistent or always clearly distinguishable across subjects, the condition effects are examined by averaging response amplitudes within the specified latency windows. The first average deflection peaking at 140 ms (average amplitude within 120–150 ms) is not differentiated by any of the stimulus characteristics, as indicated by the lack of main effect, *F*_(1, 13)_ = 0.1, ns (Figures 2, 3). However, the main effect of the deflection peaking at 180 ms (average amplitude within 170–190 ms latency window), *F*_(5, 65)_ = 2.5, *p* < 0.05, reflected its sensitivity to inversion. This deflection tends to be greater to inverted than all other stimuli, *F*_(1, 13)_ = 4.0, *p* < 0.07 (uncorrected). A similar pattern but with a more robust effect of inversion is observed in the left aTL at this latency (Figure 2), with a significant main effect of condition, *F*_(5, 65)_ = 9.6, *p* < 0.001. In the aTL, inverted faces elicit greater activity than all other stimuli at ~180 ms, *F*_(1, 13)_ = 22.5, *p* < 0.001. Therefore, inverted faces selectively engage the left ventral temporal cortex with slightly longer peak latency than the right-dominant fusiform area.

The M160 is followed by another peak at ~240 ms (M240) after stimulus onset (Figures 1–3). The strongest M240 is elicited by normal faces, especially along the ventral stream, including the left FG and aTL bilaterally. ANOVA of the peak amplitudes within 210–250 ms latency in the left FG revealed a main effect of condition, *F*_(5, 65)_ = 2.5, *p* < 0.05, with a tendency for normal faces eliciting greater activity than all other stimuli, *F*_(1, 13)_ = 8.4, *p* < 0.07. In the left anterior ventrolateral temporal cortex, the activity to normal faces was also stronger than to all other stimuli overall, *F*_(1, 13)_ = 11.4, *p* < 0.05, although it did not differ from the stimuli with features presented within the oval. The peak latency (239 ± 16 ms) did not differ across conditions with the exception of a longer peak latency trend for the inverted faces (246 ms), *F*_(1, 13)_ = 7.9, *p* < 0.09. Finally, as on the left, the activity to normal faces in the right aTL was greater than to other stimuli within 220–270 ms time window, *F*_(1, 13)_ = 9.8, *p* < 0.05. The peak latency (255 ± 20 ms) was longer on the right than on the left, *F*_(1, 13)_ = 36.1, *p* < 0.001.

## Discussion

Our results support models proposing that face processing unfolds in successive, but overlapping and mutually dependent spatio-temporal stages in the ventral visual stream. The incoming face stimuli are analyzed for their visual characteristics at ~100 ms in the occipital visual areas as indexed by M107. Structural encoding of the face-specific aspects takes place in the FG at ~160 ms (M160) especially on the right, with the exception of the inverted faces that additionally activate anteroventral temporal cortex on the left. Subsequent, presumably more integrative processing, engages distributed inferoventral and anterolateral temporal areas at ~240 ms (M240) bilaterally. These latencies of face-related activity peaks have been observed in other MEG studies (Schweinberger et al., 2007; Taylor et al., 2011) and confirmed with iEEG (Barbeau et al., 2008), lending further support to similar stages proposed by other models (Bruce and Young, 1986; Halgren et al., 1994a; Haxby et al., 2000).

### M107—sensitivity to low-level visual features

In the present study, the initial activity peak (M107) in the occipital area is greater to inverted and randomized control faces in comparison to other stimulus categories. Other ERP and MEG studies have also reported larger peak at ~100 ms to inverted faces (Linkenkaer-Hansen et al., 1998; Itier and Taylor, 2002, 2004a; Schweinberger et al., 2007; Meeren et al., 2008) and to randomized control faces (Halgren et al., 2000) in comparison to normal faces. Based on such findings, it has been proposed that stimulus categorization takes place at ~100 ms based on holistic perception of a face (Liu et al., 2002b; Itier and Taylor, 2004b). However, other evidence suggests that the activity differences may be merely due to low-level visual differences. MEG studies indicate that the mid-occipital M100 amplitude is increased as a function of parametrically varied pixel noise (Tarkiainen et al., 2002) and spatial frequency (Tanskanen et al., 2005). Similarly, the fMRI-BOLD signal is larger to visually randomized faces in retinotopic areas (Lerner et al., 2001). This evidence is consistent with the idea that the observed categorical differentiation at ~100 ms is based on low-level visual characteristics rather than a holistic percept (Rossion and Caharel, 2011; Cauchoix et al., 2014). Nevertheless, this deflection may represent an initial step in the face-sensitive analysis of the global visual characteristics with the purpose of tuning and facilitating subsequent processing (Halgren et al., 1994a; Itier and Taylor, 2004a). All of our stimuli belong to the face-like category, but those that deviate more from a global face template based on their shape (inverted faces) or texture and contour (randomized control stimuli) evoke the strongest M107 activity in the occipital area (Figure 2). Based on its sensitivity to low-level features, this initial stage may serve as a domain-specific gate, “flagging” stimuli that deviate in orientation or shape (Portin et al., 1999; Tsao and Livingstone, 2008) and allowing for a fast visual categorization (Crouzet and Thorpe, 2011). This stage may facilitate subsequent structural encoding stage which is represented in the FG at 160 ms, carrying out further refinement (Rossion and Caharel, 2011).

### M160—global face encoding

This stage is reflected in a strongly right-lateralized M160 deflection which was greatest to inverted faces. All other face-like stimuli (normal, oval, and rearranged) evoked similar, intermediate-level activity in the fusiform cortex, whereas blank and randomized control faces evoked the weakest activity (Figure 2). This suggests that the face representation formed at this stage is based on a roughly face-like template that contains basic visual elements of a face: oval-shaped contour in an upright position with contrasting facial features regardless of whether they are spaced appropriately. Although the M160 representation lacks precision allowing for individuation at this stage, the stimuli that were most face-like evoked stronger activity than the blank faces and control stimuli which carry very little visual information needed for subsequent recognition. Our data are consistent with previous suggestions that this deflection reflects the operation of a face-encoding processing stage (Halgren et al., 1994a; Bentin et al., 1996; Puce et al., 1999; Eimer, 2000b; Downing et al., 2001; Bentin and Carmel, 2002), akin to the structural encoding (“face detection”) module originally proposed by Bruce and Young (1986). In contrast to M107 which is sensitive to gross visual characteristics, the M160 deflection (presumably analogous to N170 in the ERP literature) is larger to stimuli that broadly resemble faces and can be processed further for familiarity. Consistent with other evidence, the M160 is responsive to the presence of facial features in the veridical or rearranged configuration irrespective of the facial outline (Bentin et al., 1996; Zion-Golumbic and Bentin, 2007). The M160 is attenuated to blank faces that lack internal features and to randomized control stimuli, confirming other similar findings at this latency in the FG (Eimer, 2000b; Tong et al., 2000). The finding that the right-lateralized M160 is similar in amplitude to stimuli containing inner features irrespective of their configuration could represent a process broadly generalizable to other types of visual stimuli such as words. For instance, ventral temporal cortex on the left is comparably activated by real and pseudowords, but not by other control stimuli (Cohen et al., 2002). In other words, the presence of the requisite features even if they are in unnatural locations may be necessary and sufficient for initial acceptance of a stimulus as possibly representing a face. This aspect of the face processor may be useful in situation when faces are seen in non-habitual orientations (for example, when the observed face is on a person lying on her side) and/or when much of the face is obscured by a hat or hair).

The N170 is largely insensitive to familiarity or repetition and consequently unresponsive to individuation (Marinkovic and Halgren, 1998; Puce et al., 1999; Bentin and Deouell, 2000; Eimer, 2000a; Anaki et al., 2007; Schweinberger et al., 2007; Barbeau et al., 2008; Taylor et al., 2011; Rivolta et al., 2012), providing additional evidence for its role in global face encoding (Bentin et al., 1996). In contrast, the process of individuation and recognition is subserved at the subsequent stage at ~240 ms, located downstream in temporal cortices bilaterally. During the M160, the face-like features may be extracted by a domain-specific mechanism, permitting formation of a unitary and holistic representation of a face (Tanaka and Farah, 1993; Bentin and Golland, 2002; Schiltz and Rossion, 2006; Jacques and Rossion, 2009). This representation may be projected to distributed association cortices for further mnemonic, semantic, and emotional processing, resulting in the integration of the recognition process, as suggested by face-selective broadband coherence in intracranial EEG between the fusiform and distributed cortical areas (Klopp et al., 2000). The M160 was estimated to the right-dominant ventral temporal area, in the FG. Indeed, intracranial recordings confirm that the primary generators of the N170 deflection are in the fusiform area (Allison et al., 1994; Halgren et al., 1994a; McCarthy et al., 1997; Puce et al., 1997; Barbeau et al., 2008), in agreement with neuroimaging evidence (Kanwisher and Yovel, 2006).

### Face inversion engages dual-route processing

The M160 in the right fusiform cortex to inverted faces had a larger amplitude and longer peak latency than all other stimuli, replicating results of numerous other ERP and MEG studies (Bentin et al., 1996; Eimer, 2000a; Rossion et al., 1999, 2000; Liu et al., 2000; Sagiv and Bentin, 2001; Itier and Taylor, 2002, 2004a; Watanabe et al., 2003; Kloth et al., 2006; Honda et al., 2007). In the left ventral temporal cortex, the immediately preceding deflection peaked at ~140 ms and was insensitive to any manipulation (Figures 2, 3). However, the immediately subsequent deflection peaking at ~180 ms on the left was selectively elicited by inverted faces (Figure 2) in a manner similar to the right M160. Clearly, the M160 is not maximal to optimal stimuli (i.e., normal, upright faces) but to inverted stimuli that deviate from the canonical orientation. At this point, the inverted faces have been classified as faces and need to engage additional resources to continue being processed for recognition. Even though at this latency the overall activity is much weaker in the LH overall, the deflection at 180 ms is elicited selectively by inverted faces. This indicates that they may uniquely engage bilateral ventral temporal cortices, supporting a dual route model (Moscovitch et al., 1997; De Gelder and Rouw, 2001; Rhodes et al., 2004), as well as the related idea that inverted faces recruit other mechanisms in addition to the right fusiform region (Aguirre et al., 1999; Haxby et al., 1999; Rossion et al., 2000; Yovel and Kanwisher, 2005; Epstein et al., 2006; Rossion, 2009). Despite a clear RH dominance in face processing, some evidence suggests that the LH contributes significantly to processing inverted faces. Behavioral studies using divided visual field methodology show the RH advantage in discriminating upright, but not inverted faces (Hillger and Koenig, 1991; Cattaneo et al., 2013), indicating left hemisphere engagement during processing of inverted faces. Similarly, split-brain monkeys show the face inversion effect when the stimuli are presented to the RH, but not to the LH (Vermeire and Hamilton, 1998). The face recognition deficit in prosopagnosic patients is more pronounced with bilateral lesions (Barton, 2008), possibly resulting from a disruption in interhemispheric communication which is critical for integrated perceptual decisions. Furthermore, relatively spared processing of inverted faces in prosopagnosia (Farah, 1996; De Gelder and Rouw, 2001) could be explained by a model of bilateral engagement of a more general system for visual objects (Aguirre et al., 1999; Haxby et al., 1999). Finally, MEG studies (Dobel et al., 2008, 2011) reported that individuals with congenital prosopagnosia manifested a decreased M170 and a strongly reduced gamma power in the left fusiform cortex, confirming left hemisphere involvement in normal face processing. This observation is confirmed by an fMRI study showing decreased activation in the left FG in congenital prosopagnosic patients (Dinkelacker et al., 2011). Therefore, it appears that by disturbing canonical face processing, face inversion creates suboptimal conditions for face recognition (Rossion, 2008), resulting in bilateral engagement of the ventral visual stream. This effect is not unique inasmuch as the N170 is similarly augmented to contrast inversion and misaligned face halves (Itier and Taylor, 2002; Letourneau and Mitchell, 2008; Jacques and Rossion, 2010) which may also rely on additional visual processing mechanisms. Furthermore, engagement of additional resources in the non-dominant hemisphere by visually deviating stimuli may be a more general principle generalizing beyond faces. For instance, even though left-dominance of language processing has been firmly established (Price, 2010), the right hemisphere is selectively engaged by unpronounceable non-words (Marinkovic et al., 2014). Similarly, the right ventral occipitotemporal cortex is more strongly activated by words in the less fluent language in bilingual speakers (Leonard et al., 2010).

### M240—emergence of familiarity via repetition

Extensive imaging evidence obtained with hemodynamic methods has been commonly interpreted in the context of dedicated face-processing modules particularly in the fusiform area (Kanwisher et al., 1997; Kanwisher and Yovel, 2006). However, spatio-temporally sensitive methods impose the idea of distributed and partly sequential processing encompassing mutually dependent and overlapping areas whereby the face-relevant information is increasingly refined in the posterior-to-anterior direction, reaching identity/semantic networks in the anterior temporal and inferior prefrontal cortices (Halgren et al., 1994a,b, 2000; Puce et al., 1999; Barbeau et al., 2008). Faces are processed by the ventral processing stream similar to other visual stimuli. Subsequent to an early engagement of the striate cortex (M107), ventral occipito-temporal areas support an intermediate material-specific processing stage (M160) providing structural representations to downstream distributed associative areas for processing of identity and emotional expression (Bruce and Young, 1986; Klopp et al., 2000; Liu et al., 2002b). In contrast to M107 and M160 that were larger to inverted faces, the normal, upright faces evoked the largest M240 estimated to the ventral and anterior temporal areas bilaterally, in agreement with other MEG reports (Schweinberger et al., 2007). The M240 deflection engages distributed anterior temporal cortices and may index familiarity detection and recognition, supporting previous iEEG findings (Barbeau et al., 2008). Furthermore, recent evidence shows that the (presumably analogous) N250 is sensitive not only to familiarity (Caharel et al., 2014), but that it emerges to previously unfamiliar faces as a result of repetition and, consequently, familiarization (Tanaka et al., 2006; Schweinberger et al., 2007; Pierce et al., 2011; Zimmermann and Eimer, 2013). Even though we did not manipulate repetition in a condition-specific manner, the present results are consistent with the idea that this deflection may reflect access to recognition units and activation of a memory trace for the particular face that has become familiar with repetition (Zimmermann and Eimer, 2013). Our localization estimates and the observation of the sensitivity of the inferior and anterior temporal cortices to face orientation and identity are supported by fMRI studies (Sugiura et al., 2001; Rotshtein et al., 2005; Kriegeskorte et al., 2007; Nasr and Tootell, 2012; O'Neil et al., 2013) and are further confirmed with single cell recordings in non-human primates (Freiwald and Tsao, 2010). Similarly, lesion studies report that anterior temporal lesions result in face recognition impairments (Glosser et al., 2003; Barton, 2008; Gainotti and Marra, 2011). Thus, it appears that familiarity detection stage depends on the anterior temporal structures, and possibly specifically perirhinal cortex (Allison et al., 1994; Halgren et al., 1994a; Henson et al., 2003).

Even though the estimated M240 sources in our study are bilaterally distributed, the overall activity is left-dominant. It is generally accepted that the left hemisphere is essential for semantic domain especially in language tasks whereas the right hemisphere subserves face processing (Dien, 2009). Right hemisphere bias for faces has been widely reported and accepted (De Renzi et al., 1994; Kanwisher et al., 1997). However, even during face processing left hemisphere may play a dominant role in storage and retrieval of semantic face attributions as indicated by lesion (Glosser et al., 2003; Snowden et al., 2004) and imaging evidence (Griffith et al., 2006). Given that in our study only photographs of previously unknown faces were used, the connection with semantic system is speculative. Nevertheless, an increase in M240 resulting from repeated exposure to upright, normal faces may partially stem from initial engagement of the network supporting person-specific information (Gainotti and Marra, 2011; Zimmermann and Eimer, 2013). These semantic face attributions may be represented in the left hemisphere as is the case with left-lateralized N360 to famous faces (Barbeau et al., 2008). Baron and Osherson (2011) used face stimuli in a visual categorization task and showed that the left anterior temporal lobe was especially sensitive to combinatorial face categorization. Importance of the left hemisphere is supported by reports of prosopagnosia resulting from ventral lesions in the left hemisphere (Verstichel and Chia, 1999; Vuilleumier et al., 2003). Furthermore, Dinkelacker et al. (2011) showed decreased fMRI activation in the left FG in congenital prosopagnosic individuals. Similarly, a MEG study found weaker activity overall in the left occipitotemporal areas in congenital prosopagnosic patients (Dobel et al., 2008). Nevertheless, the overwhelming evidence suggests that face processing depends on distributed bilateral contributions (Farah, 1990; Haxby et al., 2000; Verosky and Turk-Browne, 2012) even in the case of emotional face processing (Fusar-Poli et al., 2009).

The anterolateral and ventral temporal regions may be essential for bringing together the configural representation of the face stimuli with the identity-relevant representations as part of a distributed network (Avidan et al., 2013; O'Neil et al., 2014). iEEG recordings show coherence between the FG and distributed association areas at ~200 ms to faces (Klopp et al., 2000) and functional connectivity studies support this finding (O'Neil et al., 2014). This transitional entrainment may represent a widespread projection for further processing. The M240 may thus represent the familiarity detection stage as an initial step in accessing the person identity/semantic system that exists for the famous faces or personal acquaintances, followed by the full-fledged recognition percept laden with emotional, mnemonic, and other associations (Halgren et al., 1994a). Since the participants in our experiment were engaged in passive viewing of unfamiliar faces and were not asked to make any explicit judgments, we interpret the M240 as an index of familiarity with caution. Nevertheless, people excel at making attributions about unfamiliar faces such as age, gender, attractiveness, intelligence, etc. (Bruce and Young, 1986) and the M240 may index a familiarity detection stage within a generic face processing stream.

## Conclusion

Faces are highly relevant visual objects engaging a multi-stage cascade of mutually dependent and overlapping distributed activity in the ventral visual stream with flexible downstream allocation. An initial analysis of the low-level visual characteristics takes place in the occipital region at ~100 ms. Its sensitivity to low-level visual features and deviation in orientation or shape and texture may facilitate fast initial categorization. The subsequent activity of the predominantly right ventral temporal area (centered on the posterior FG) at ~160 ms may index the face detection stage by subserving structural encoding necessary for downstream individuation and recognition. Additional engagement of the left ventral temporal area at ~180 ms by inverted faces is consistent with the dual route model and spared processing of inverted faces in prosopagnosia. The M240 may index engagement of the familiarity processing network in bilateral, distributed anteroventral temporal areas. Thus, our data support dynamic models of face processing that suggest that face perception is subserved by a distributed and interactive neural circuit (Bruce and Young, 1986; Halgren et al., 1994a,b; Puce et al., 1999; Haxby et al., 2000; Klopp et al., 2000; De Gelder and Rouw, 2001; Rossion et al., 2003b; Ishai et al., 2005; Barbeau et al., 2008; Nasr and Tootell, 2012; Cauchoix et al., 2014).

### Conflict of interest statement

The authors declare that the research was conducted in the absence of any commercial or financial relationships that could be construed as a potential conflict of interest.
